# Treatment of Naturally Occurring Tendon Disease with Allogeneic Multipotent Mesenchymal Stromal Cells: A Randomized, Controlled, Triple-Blinded Pilot Study in Horses

**DOI:** 10.3390/cells12212513

**Published:** 2023-10-24

**Authors:** Janina Burk, Liza Wittenberg-Voges, Susanna Schubert, Carolin Horstmeier, Walter Brehm, Florian Geburek

**Affiliations:** 1 Institute of Physiology, Pathophysiology and Biophysics, University of Veterinary Medicine Vienna, Veterinaerplatz 1, 1210 Vienna, Austria; 2 Clinic for Horses, University of Veterinary Medicine Hannover, Foundation, Bünteweg 9, 30559 Hannover, Germany; liza.wittenberg-voges@tiho-hannover.de; 3 Institute of Human Genetics, University of Leipzig Medical Center, Philipp-Rosenthal-Strasse 55, 04103 Leipzig, Germany; susanna.schubert@medizin.uni-leipzig.de; 4 Department for Horses, Veterinary Teaching Hospital, University of Leipzig, An den Tierkliniken 21, 04103 Leipzig, Germany; caro.horstmeier@web.de (C.H.); brehm@vetmed.uni-leipzig.de (W.B.)

**Keywords:** tendinopathy, tendon lesion, mesenchymal stem cells, mesenchymal progenitor cells, MSC, adipose, horse serum, clinical study, re-injury, recurrence rate

## Abstract

The treatment of tendinopathies with multipotent mesenchymal stromal cells (MSCs) is a promising option in equine and human medicine. However, conclusive clinical evidence is lacking. The purpose of this study was to gain insight into clinical treatment efficacy and to identify suitable outcome measures for larger clinical studies. Fifteen horses with early naturally occurring tendon disease were assigned to intralesional treatment with allogeneic adipose-derived MSCs suspended in serum or with serum alone through block randomization (dosage adapted to lesion size). Clinicians and horse owners remained blinded to the treatment during 12 months (seven horses per group) and 18 months (seven MSC-group and five control-group horses) of follow-up including clinical examinations and diagnostic imaging. Clinical inflammation, lameness, and ultrasonography scores improved more over time in the MSC group. The lameness score difference significantly improved in the MSC group compared with the control group after 6 months. In the MSC group, five out of the seven horses were free of re-injuries and back to training until 12 and 18 months. In the control group, three out of the seven horses were free of re-injuries until 12 months. These results suggest that MSCs are effective for the treatment of early-phase tendon disease and provide a basis for a larger controlled study.

## 1. Introduction

The multipotent mesenchymal stromal cell (MSC) treatment of tendon injuries in horses was first described in a report dating almost 20 years back by now [1]. Since this pioneering case study, MSCs received tremendous attention in the equine community [2,3,4,5,6,7,8], and hope was raised that translation into human therapies could be facilitated based on experiences in equine medicine. In the following years, large case studies were published with highly promising outcomes [9,10], albeit with historical or non-randomized [11] controls, which were not directly comparable. Furthermore, several studies were performed that focused on the treatment of experimentally induced equine tendon injuries with MSCs. Most of them reported improvements in the tendon structure and composition [12,13,14,15], but this could not be convincingly confirmed in other experimental studies [16,17].

The conflicting outcomes of experimental studies could be due to different models for tendon lesion induction used (collagenase injection vs. mechanical disruption), while no model can actually reflect the complex naturally occurring pathophysiology [18,19,20,21]. In this context, the study in horses with naturally occurring tendinopathy performed by Smith et al. (2013) needs to be highlighted [22]. This study confirmed the improvement in histological, biochemical, and biomechanical parameters upon MSC treatment as compared to controls in a more clinically relevant context. However, the animals were sacrificed instead of being returned to full training in that study; thus, no information on long-term clinical outcomes was available. Furthermore, the horses were treated with autologous bone marrow-derived MSCs in bone marrow supernatant, while the controls received saline [22], which did not allow for conclusions regarding the efficacy of the MSCs alone. Eventually, a decade later, it must be acknowledged that the clinical evidence of improved healing after local MSC treatment is still not sufficient.

The lack of a comprehensive clinical study on horses is due to the anticipated difficulties in the recruitment of suitable equine patients and compliant horse owners who agree to the blinded treatment and cope with the efforts associated with the follow-up [11]. Nevertheless, this challenge has to be addressed to reach a satisfactory level of evidence for this MSC-based regenerative therapy. Consequently, the authors performed a prospective, randomized, controlled, and triple-blind pilot study, aiming to establish the prerequisites for the design of a larger study that can finally provide evidence with a simplified, efficient follow-up, given the fact that the outcome of this pilot study would justify further efforts.

## 2. Materials and Methods

### 2.1. Aims and Study Design

The aims of this study were to gain first insights into the efficacy of local allogeneic adipose-derived MSC treatment for naturally occurring tendon disease and to identify suitable outcome measures for tendon healing in this context. Equine patients were recruited at three German veterinary teaching hospitals, examined, and allocated to treatment groups through stratified block randomization. Strata were defined based on the duration of symptoms before the first presentation (A: <2 weeks; B: 2 weeks to 2 months). Treatment included the intralesional injection of MSCs in horse serum (MSC group) or horse serum only (control), as allocated by the laboratory staff. Standardized clinical examinations and diagnostic imaging were performed before treatment and 6 weeks as well as 3, 6, 12, and 18 months after treatment. All horses were subjected to a controlled exercise program. The rate of re-injuries until 12 months after treatment was chosen as the primary endpoint. Veterinarians, examiners, and horse owners were blinded to the treatment group.

### 2.2. MSC Recovery and Culture

The MSCs had been isolated using an enzyme-free explant culture from a healthy donor warmblood horse (gelding, aged 6 years) euthanized for unrelated reasons. The cells were isolated and expanded without xenogeneic serum supplements. A StemMACS™ MSC Expansion Media Kit XF human (Miltenyi, Bergisch Gladbach, Germany) was used as the culture medium, and 1% penicillin–streptomycin and 0.1% gentamycin were added during the explant culture. TrypLE™ Express Enzyme (Gibco™, ThermoFisher Scientific, Dreieich, Germany) was used for passaging. After the first passaging, CD14+ cells were depleted via magnetic cell separation as detailed previously [23]. The MSCs were then further expanded and split into portions for cryopreservation at passage 2, using a Synth-a-Freeze™ cryopreservation medium (Gibco™, ThermoFisher Scientific, Dreieich, Germany) and a Mr. Frosty freezing container (Nalgene™, ThermoFisher Scientific, Dreieich, Germany) according to the manufacturer’s instructions. For MSC treatments, MSCs were thawed and expanded in the same culture medium but supplemented with 2% horse serum (Sigma-Aldrich, Taufkirchen, Germany) and without antibiotics. After cell harvest at passage 3, MSCs were suspended in the same horse serum (5 × 10^6^ MSCs per mL) and aspirated in opaque syringes for transport and cell injection. Syringes with serum only were prepared for the control group. Aliquots of MSCs before and after cryopreservation were subjected to MSC characterization, including immunophenotyping and differentiation into mesenchymal lineage cells. Differentiation [23]; monoclonal antibody staining for CD29, CD73, CD90, CD105, CD14, CD34, CD45, CD79α, MHCII [23], and CD44 [24]; and flow cytometric analysis [23,24] were performed as described in detail earlier. Chondrogenic differentiation was not attempted to reduce the cell numbers required for characterization. Routine quality controls regarding cell morphology and viability were performed and documented during each culture. The transport of MSCs and serum to the respective clinic was carried out overnight under controlled cooled conditions.

### 2.3. Animals and Inclusion Criteria

Horses aged between 3 and 25 years and presenting with naturally occurring tendon disease were recruited. To be included, the injury had to be located within the metacarpal region of one of the forelimb superficial digital flexor tendons, and the onset of symptoms had to date back no more than 2 months prior to initial presentation. Previous systemic treatment with non-steroidal anti-inflammatory drugs and local treatments with bandages and ointments were tolerated, whereas horses that had already received any intralesional or other than the mentioned treatments were excluded. Moreover, the owners had to agree to follow a standardized rehabilitation training schedule and to present their horses for regular follow-up examinations for at least 12 months, with an optional final examination 18 months after treatment. Fifteen horses were initially included, one of which had to be excluded later and was replaced due to significant non-compliance with the rehabilitation training schedule. Seven horses per treatment group completed the study until the primary endpoint 12 months after treatment and were included in data analyses (Table 1).

### 2.4. Treatment Procedure

The horses were treated with a local injection of either allogeneic adipose-derived MSCs suspended in GMP-grade horse serum or with this type of serum alone, as defined through block randomization. The treatment dose was adapted to the size of the tendon lesion, as determined by B-mode ultrasonography during the initial examination (5 × 10^6^ MSCs in 1 mL serum or 1 mL serum per 1 cm^3^ lesion volume). For treatment, the horses were sedated using detomidine (0.01 mg per kg bwt; Cespesedan^®^, CP-Pharma, Burgdorf, Germany) and butorphanol (0.01 mg per kg bwt; Butorgesic^®^, CP-Pharma), an ulnar nerve block was performed using lidocaine 2% (Bela-pharm, Vechta, Germany), and the injection site was clipped and aseptically prepared. After intracutaneous infiltration with 1 mL of lidocaine, MSCs and/or serum were injected intralesionally using a 19 G cannula [25] under ultrasonographic guidance. A lateral approach transverse to the longitudinal axis of the tendon was chosen while the horse was bearing weight. The maximum volume per injection site was set at 2 mL, so lesions with a volume >2 cm^3^ were injected at two or more equidistant sites to achieve a homogeneous distribution of MSCs or serum within the lesion. The distal limbs were bandaged, and the horses received phenylbutazone (2.2 mg per kg bwt; CP-Pharma) for 2 days [26] and were returned to their owners. Two days after treatment, the horses started the rehabilitation training with their owners, using a previously described controlled exercise schedule [27], which consisted of 16 weeks of walking exercise (gradually increasing intervals from 5 to 40 min), followed by 16 weeks with additional training at a trot (increasing intervals from 5 to 20 min) while intervals of walking exercise were decreased (from 40 to 25 min). During the final 16 weeks, the horses were exercised 45 min daily, including gradually increasing gallop training. The exercise schedule was adapted if necessary, depending on the cumulative clinical inflammation and lameness score. The two possible scenarios necessitating schedule adjustment were as follows:

1. If a horse was lame at a trot (score 1 or 2/5) during a scheduled examination or showed an increase in clinical signs of inflammation (increase in heat, pain, or swelling) compared with the previous examination, the exercise level was not further increased. If the horse had already been scheduled to trot or gallop, exercise was reduced to hand walking. In these cases, a clinical revaluation was performed three weeks later, and the level of exercise was determined depending on the respective clinical findings.
2. If a horse was lame at a walk (score ≥ 3/5) on the day of examination, the horse received box rest and was re-evaluated 7 days later. If lameness at walk was detected on the day of re-evaluation, the standard exercise schedule was restarted from the beginning.

Furthermore, the exercise schedule was adjusted in accordance with the owner if deemed necessary due to other pathologies (e.g., colic, lameness on another leg, etc.).

### 2.5. Clinical Follow-Up

At each examination, a cumulative clinical inflammation score of the injury site was recorded, which included the parameters of swelling, pain to palpation, and skin surface temperature, with each parameter ranging from 0 score points (normal) to 3 score points (highly abnormal) [28]. Furthermore, weight bearing and gait were evaluated during walking and trot, using a lameness score ranging from 0 (normal) to 5 (highly abnormal) [16]. This was performed on hard and soft ground, and the score points for each were added up to a total lameness score. Finally, possible adverse reactions, the progress of the rehabilitation training, and the occurrence of re-injuries were documented. Adverse reactions and re-injuries were confirmed by the examining veterinarian after being reported by the owner.

### 2.6. Diagnostic Imaging

All clinical examinations were complemented using diagnostic imaging. B-mode ultrasonography, using Logiq E9 or Logiq P2 (GE-Healthcare, Solingen, Germany) or Aplio MX (Toshiba, Zoetermeer, The Netherlands) machines, was performed in all horses. Seven transverse and four longitudinal images were acquired from the metacarpal tendon region at defined levels [29]. The findings were documented using a previously described score [30], based on which the tendon was evaluated regarding echogenicity and fiber alignment, with score points ranging from 0 (normal) to 3 (highly abnormal) for each parameter. Both scores for all images per examination were added up for further analysis. Furthermore, the tendon cross-sectional area (TCSA) was measured at each transverse level. The mean TCSA of the whole metacarpal tendon segment was calculated, and these data were normalized to the equivalent contralateral TCSA value to account for the differences in body height.

Moreover, most equine patients (n = 6 per group) additionally underwent color Doppler ultrasonography (CDU) using the ultrasound machines specified above and ultrasonographic tissue characterization (UTC; UTC scan unit, configuration 2011, UTC Imaging B.V., Stein, Netherlands). For both, again the whole metacarpal tendon segment was included in the assessment. In CDU, the mean percentage of vascularized area within the tendon was calculated from five images per examination as previously described [31]. In UTC, the percentage of altered and non-mature tendon tissue, as displayed by black (echo type IV), red (echo type III), and blue (echo type II) pixels, was calculated [16] and normalized to the contralateral tendon. Exemplary equine patients (n = 1 per group) were subject to 0.27 Tesla magnetic resonance imaging instead of CDU and UTC, using the Hallmarq Standing Equine MRI system (Hallmarq, Surrey, UK) as described previously [32].

### 2.7. Statistical Analysis

Statistical analysis was performed using SPSS Statistics 26 (IBM, Ehningen, Germany). As data were not normally distributed, non-parametric tests were used. To compare the outcomes over time with the status before treatment, Friedman and subsequent post hoc tests for paired samples were used within each group, and *p*-values were Bonferroni-corrected for the number of relevant comparisons per analysis. To compare the two groups directly, the differences in the respective parameters over time were calculated for each time point (The value before treatment, i.e., at month 0 was substracted from the value at a the respective timepoint of control), so that higher values reflect a worsening effect, and lower values indicate improvement. With these data, Mann–Whitney U tests for unpaired samples were computed for group comparisons in a hypothesis-based approach. Differences were considered as significant at *p* ≤ 0.05.

## 3. Results

### 3.1. MSC Characterization

**Figure 1:**
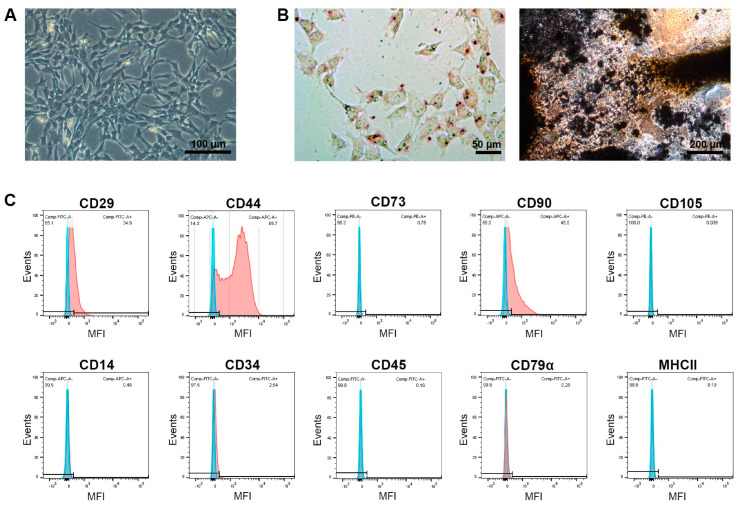
**Characterization of mesenchymal stromal cells (MSCs).** Phase-contrast microscopy displays the morphology of the undifferentiated cells (**A**), and light microscopy displays the cells after adipogenic differentiation and oil red O staining (left) and osteogenic differentiation and von Kossa staining (right) (**B**). The histograms display the flow cytometric analysis of the MSCs after monoclonal antibody staining of the respective marker antigens (red) and corresponding isotype controls (blue) (**C**). The images and data shown here were obtained from MSCs after thawing and expansion, corresponding to the cells used for treatment.

The MSCs were spindle-shaped in plastic-adherent culture and capable of differentiation into adipocytes and osteoblasts, as evidenced by lipid vacuole accumulation and the deposition of mineralized matrix, respectively. This was confirmed after cell isolation and sorting as well as for exemplary MSC portions that had been thawed, expanded, and used for treatment. After isolation and sorting, the MSCs were positive for the inclusion markers CD29, CD44, and CD90 (87%, 99%, and 75% positive cells, respectively) but widely negative for CD73 and CD105 (1% and 6%, respectively). They also showed very low expression of the exclusion marker antigens CD14, CD34, CD45, CD79α, and MHCII (2%, 4%, 5%, 0%, and 3%, respectively). The immunophenotype remained similar after thawing and further expansion, albeit with overall decreased percentages of positive cells. An exemplary analysis of the MSCs used for treatment (i.e., after thawing and expansion) is shown in Figure 1.

### 3.2. Animals and Tendon Lesions Included

In total, 14 equine patients (n = 7 per group) reached the defined primary endpoint 12 months after treatment and were used for data analysis. Two horses in the control group did not present for the last examination 18 months after treatment, as decided by their owners. Out of the total 14 analyzed horses, 2 per group presented with acute lesions (onset of symptoms <2 weeks), and 5 per group presented with early lesions (onset of symptoms between 2 weeks and 2 months). The duration of symptoms tended to be longer, and lesions tended to be larger in the MSC group. Furthermore, the horses in the MSC group tended to be younger. However, these differences were small, and none of them was significant (Table 1).

### 3.3. Clinical Findings

No severe adverse reactions to the treatment were reported. A transient increase in the lesion was observed in one horse in the MSC group, which still showed good tendon healing during the course of the study. The exercise schedule was adjusted in nine cases due to a lack of improvement or worsening in the clinical inflammation and lameness scores. In one additional case, the level of exercise was reduced because the horse needed a ventral midline laparotomy (colic surgery) during the study period and could only be hand-walked for the following 6 weeks.

**Figure 2:**
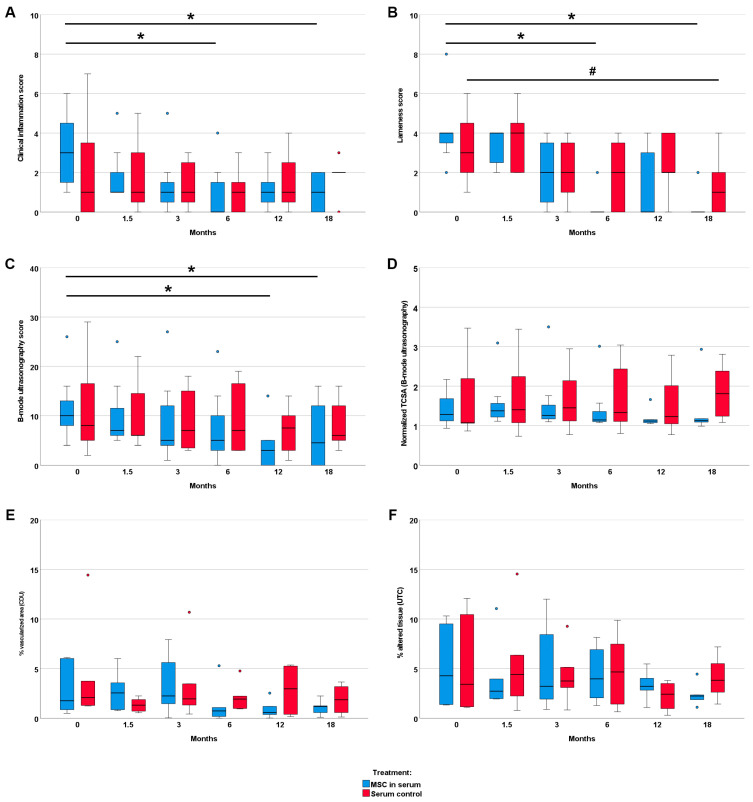
**Clinical and image parameters.** Follow-up examinations were performed over a time period of 18 months after treatment of early naturally occurring equine tendon lesions with either allogeneic mesenchymal stromal cells (MSCs) in serum or serum alone. Clinical inflammation scores (**A**) and lameness scores (**B**) were obtained using blinded clinical examinations. The B-mode ultrasonography score (**C**), the normalized TSCA (tendon cross-sectional area) (**D**), the % vascularized area (**E**), and the normalized % altered tissue (echo types II, III and IV) (**F**) were obtained through the blinded (semi)quantitative analysis of ultrasound-based imaging (CDU: color Doppler ultrasonography; UTC: ultrasound tissue characterization). The 0-month examinations were performed directly before the horses were treated with MSCs and/or serum. The asterisks (*) mark significant differences over time in the MSC group, and the hashtag (#) indicates significant differences in the serum control group (*p* < 0.05; Friedman tests and Bonferroni-corrected post hoc tests).

The clinical inflammation score showed a decrease over time only in the MSC group (*p* < 0.05 at 6 and 18 months, compared with the first examination). In contrast, no improvement in the clinical signs of inflammation was observed in the control group. Similarly, gait disorder and weight bearing showed more improvement in the MSC group than in the control group: In the MSC group, the lameness score significantly decreased by 6 and 18 months after treatment compared with the first examination (*p* < 0.05). In the control group, the decrease in lameness score was only significant after 18 months (*p* < 0.05) (Figure 2). The lameness score difference between 6 months after treatment and before treatment revealed a significant improvement in the MSC group as compared to the control group (*p* < 0.05) (Figure S1).

### 3.4. Diagnostic Imaging Findings

**Figure 3:**
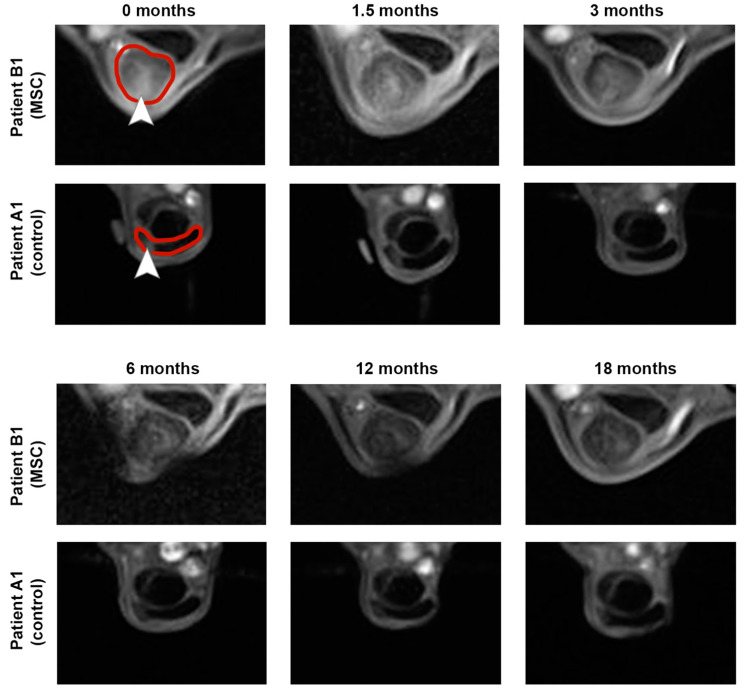
**Magnetic resonance imaging** was performed over a time period of 18 months after treatment of early naturally occurring equine tendon lesions with either allogeneic mesenchymal stromal cells (MSCs) in serum or serum alone. The transverse images show the maximum lesion level and were obtained using a T1-weighted gradient echo sequence in a 0.27 T low-field scanner. The 0-month images were acquired directly before the horses were injected with MSCs and/or serum. Both horses examined remained free of re-injuries until the end of the study, corresponding to the decreasing signal intensity within the superficial digital flexor tendons and lesion areas. The respective tendons are marked by the red line, and lesions are marked by arrowheads within the 0-month images.

Imaging findings consistently supported the clinical findings. In B-mode ultrasonography, the improvement in echogenicity and fiber alignment score was more evident in the MSC group (*p* < 0.05 at 12 and 18 months) than in the control group. B-mode ultrasonography also revealed that the mean TCSA remained widely constant in the MSC group, with a slight decrease over time, while it showed an increasing trend over time in the control group. The vascularization of the tendon, assessed via CDU, tended to be lower in the MSC group in the examinations after 6 months and later follow-ups but was transiently higher at the examination 6 weeks after treatment. The normalized percentage of altered tendon tissue, assessed via UTC, tended to decrease in the MSC group but not in the control group. However, differences in CDU and UTC were not significant. Data are presented in Figure 2 and Figure S1. Exemplary MRI images documenting the long-term healing process in two horses with no re-injuries are shown in Figure 3.

### 3.5. Re-Injury Rates and Return to Training

**Figure 4:**
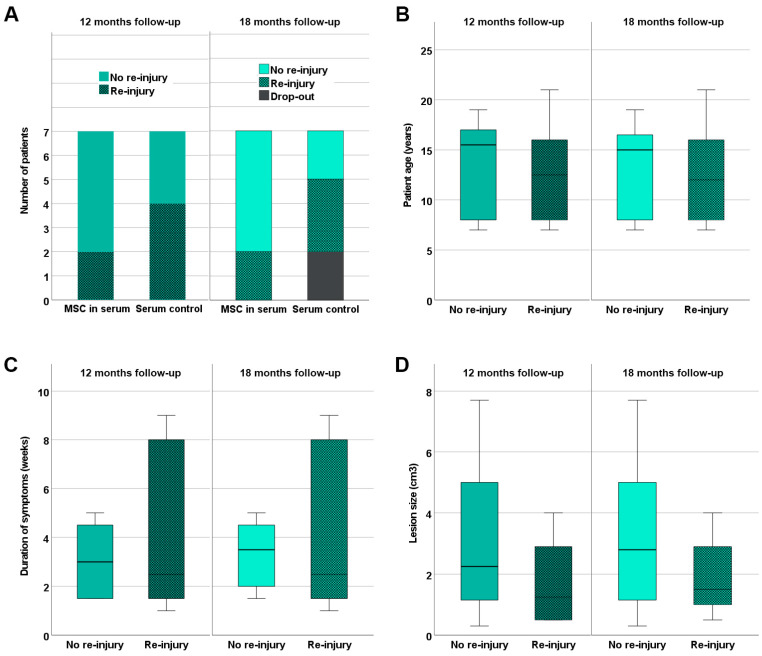
**Re-injury rates.** Re-injuries occurring until 12 and 18 months after treatment of early naturally occurring equine tendon lesions with either allogeneic mesenchymal stromal cells (MSCs) in serum or serum alone were documented. Panel (**A**) shows the number of equine patients without and with re-injury in the MSC and control groups. In the boxplot panels (**B**,**C**), horses without and with re-injury are summarized irrespective of the treatment group; their age (**B**), the duration of symptoms before inclusion in the study (**C**), and the tendon lesion size as determined with B-mode ultrasonography (**D**) are displayed. The differences observed between equine patients without and with re-injury were not significant (Mann–Whitney U test).

Five out of the seven horses in the MSC group were back in training including gallop after 12 months of follow-up, without having suffered a re-injury. Moreover, these horses remained free of recurrence until the end of the follow-up at 18 months (Figure 4). The two remaining horses in the MSC group, which included one horse that had already presented with a re-injury (animal B3), suffered a re-injury (or recurrent re-injury in the case of animal B3) before the examination 12 months after treatment, and the rehabilitation training was reduced.

In the control group, three out of the seven horses were back in training including gallop without having suffered a re-injury until 12 months after treatment. Two of them were still free of re-injuries 18 months after treatment; the third was not available for follow-up anymore. The remaining four horses in the control group had suffered a re-injury during the period of 12 months after treatment. Out of these, one horse presented with a second re-injury 18 months after treatment, and one was no longer available for the 18-month examination (Figure 4).

Comparing the re-injured equine patients to those without re-injury 12 or 18 months after treatment, no significant differences existed regarding patient age, duration of symptoms at presentation, or lesion size (Figure 4).

## 4. Discussion

Here, the results of a randomized, controlled, triple-blind pilot study on MSC treatment of equine tendon disease are presented. To the best of our knowledge, this is the first controlled study investigating the efficacy of allogeneic MSCs in naturally occurring tendon disease objectively and with long-term follow-up during rehabilitation training. Despite a relatively small number of cases included, our findings point to an improved clinical outcome after MSC treatment compared with serum treatment alone.

The MSCs used in the current study were obtained from the adipose tissue of a single healthy donor animal. Adipose tissue was chosen as an MSC source based on the previous experimental studies of our groups [16,17,23]. Allogeneic equine MSCs have repeatedly been reported to be clinically safe [33,34,35,36,37]. Nevertheless, their immunogenicity has been demonstrated and is receiving growing attention [35,37,38,39,40], and repeated injections should be avoided based on the current data [41]. Still, for single injections, the advantages of allogeneic MSC therapy are obvious, as a constant quality level of the cellular therapeutic agent can be ensured, and off-the-shelf delivery shortens the time interval between first presentation and regenerative treatment [42,43]. For MSC isolation and culture procedures, we integrated the advances made in recent years to obtain a safe and potent therapeutic agent. Firstly, xenogeneic substances were omitted as far as possible. Specifically, we completely avoided the use of fetal bovine serum, the remnants of which have by now been shown to induce the immune targeting of MSCs by the recipient, due to existing vaccination-induced anti-bovine titers [44]. However, the components of the human xeno-free StemMACS™ MSC expansion medium supplement remain undisclosed and may contain human recombinant growth factors. An equivalent for the equine species is not available on the market, and equine platelet-based alternatives [45] were still in their infancy when the current study was started [46,47]. Therefore, we considered the use of a human xeno-free medium, supplemented with equine serum, as the most reasonable choice in terms of MSC quality and safety. The cell isolation and culture procedures further involved the enzyme-free isolation of the cells using an explant technique to avoid unnecessary manipulation [48], and the depletion of CD14+ cells at first passaging. The latter was performed preventively since in previous preliminary experiments, we had sometimes observed higher percentages of CD14+ cells than allowed [49] after equine MSC culture in the StemMACS™ MSC expansion medium [23]. Finally, as we had already performed in-depth in vivo studies on the MSC fate in equine tendon lesions in the past [50,51], we avoided any cell labeling here in order to not manipulate the cells unnecessarily.

The equine patients were enrolled in the study at three different university hospitals, to increase the chance of recruiting a sufficient number of suitable cases within a reasonable time frame. Treatment and follow-up were always performed according to standardized procedures and by veterinarians specifically trained for the study, to minimize variation between locations. However, despite defined inclusion criteria, the horses recruited and their tendon lesions were a source of variation and heterogeneity. While differences in the duration of symptoms and lesion size could be acknowledged through block randomization and by adapting the dosage, not all factors with potential impact on tendon healing could be leveled out actively. These include breed and intended use of the horse as well as the localization and extent of the tendon lesion within the metacarpal region. Furthermore, compliance with the rehabilitation training schedule was a subject of interrogation during the follow-up examinations but not specifically traceable. While these factors make the current study setting representative of clinical reality, they still represent a limitation of this study and likely contributed to the observed variation in the data.

As in previous experimental trials [16,17], the horses were either treated with adipose-derived MSCs suspended in horse serum or with horse serum alone, the latter serving as the control group. This decision was made because we aimed to dissect the effect of the presence of MSCs alone and not the possible additional effects of the serum used as the vehicle. Saline was not used as the delivery vehicle or control, as it was previously shown that MSCs suspended in phosphate-buffered saline had a lower viability than MSCs suspended in bone marrow supernatant or plasma after the storage time necessary for transportation [52]. Furthermore, blood products such as autologous conditioned serum display beneficial effects on tendon healing [28], and we aimed to increase owner compliance by not including a fully untreated group.

The clinical follow-up demonstrated that signs of inflammation were significantly decreased and weight bearing, i.e., the lameness score, improved 6 and 18 months after the injection of MSCs, whereas little to no improvement was observed in the control group. The imaging findings showed similar tendencies, with a significant improvement in the B-mode ultrasonography score. Moreover, fewer re-injuries occurred in the MSC group. However, when comparing the two groups based on the difference in the respective parameters over time, differences were only significant for the lameness score 6 months after treatment, which is likely due to the variation in the data and the limited number of equine patients included in the current pilot study.

The improvement observed in tendon structure using B-mode ultrasonography corresponds to findings of previous experimental MSC studies with the treatment of collagenase-induced or naturally occurring tendon lesions [12,13,22]. In contrast, no superior effects of MSC treatment versus serum treatment alone had been observed in a previous experimental equine trial using autologous MSCs from adipose tissue in mechanically induced tendon lesions [16]. This underlines the remarkable differences between different experimental models and clinical trials with regard to the timing of treatment and the local lesion environment to which the MSCs are exposed.

In this respect, it is important to attempt a comparison of the overall outcome, i.e., the re-injury rates, in the current study to previous clinical studies including larger cohorts but with historical or non-randomized controls instead of a randomized design [9,11]. In contrast to the aforementioned experimental studies, the similarity between these investigations and the current study is that the included equine patients were returned to training and their intended use. In that context, the re-injury rates are a central parameter to evaluate the outcome [53]. When attempting to calculate the re-injury rates despite the small number of cases included here, these amount to 29% (two out of seven) in the MSC group and 57% (four out of seven) in the control group. This corresponds well to the previous findings after the MSC treatment of equine patients [9]. Thereby, the current—albeit preliminary—findings confirm the results of previous case studies with historical controls in a controlled clinical study design for the first time.

Considering the possible mode of action of the MSC treatment of early tendon disease [54], the current findings, which include a reduction in clinical signs of inflammation, can be interpreted as long-term signs of an effective immunomodulatory action [55,56]. In tendons, this effect was shown to be mediated by macrophages [57] and also appears to be associated with a transient increase in vascularization and perfusion [58]. Increased blood perfusion for a limited period of time is a vital criterion of an effective inflammatory and early proliferative phase of wound healing, but a persistent increase in perfusion is considered a sign of impaired tendon healing [59,60,61]. While MSCs, and especially those derived from adipose tissue, are known to enhance vascularization per se [62], it is critical for tendon healing that this effect is self-limiting. Indeed, in the current study, CDU signal had increased in the MSC group by 6 weeks after treatment but then decreased to lower values than observed before treatment by 6 months and later. In the control group, a contrary trend was observed. This current finding correlates well with experimental results in horses showing an increased CDU signal within the first weeks after the injection of autologous adipose-derived MSCs [31].

The early transient increase in blood perfusion and the long-term decrease in inflammation could provide a basis for improved tissue regeneration. In tendons, the latter strongly depends on the restoration of the extracellular matrix composition and architecture [63,64], which is required for biomechanical strength [65] and thus for preventing re-injuries upon mechanical loading during training. To evaluate tissue regeneration, UTC belongs to the most objective modalities for the longitudinal assessment of tendon lesions in horses [66]. However, in the current study, UTC did not yield significant differences between groups or over time. This could be due to the high sensitivity of the UTC technique [67], which strongly reflected the variation between individual lesions already at the time before treatment. Focusing on selected imaging modalities and reducing the number of examinations would reduce the efforts for the follow-up of single patients and thereby facilitate recruitment and follow-up of larger cohorts.

## 5. Conclusions

The current pilot study consistently points to improved tendon healing after MSC treatment while using a randomized, controlled, and triple-blind study design for the first time in this context. The findings strongly encourage further advanced study while providing ideal prerequisites for such future work.

## Figures and Tables

**Table 1:** Equine patients with completed follow-up for at least 12 months.

Horse Number	Group	Age (Years)	Sex	Duration of Symptoms at First Presentation	Lesion Size (cm^3^)
A1	Control	7	gelding	1.5 weeks	0.3
A2	Control	17	mare	1.5 weeks	1.7
A3	MSC	12	gelding	1 week	2.9
A4	MSC	17	gelding	1.5 weeks	1.4
B1	MSC	16	gelding	5 weeks	2.8
B2	Control	16	gelding	8 weeks	4.0
B3	MSC	8	mare	9 weeks (+acute re-injury)	1.0
B4	Control	21	mare	2.5 weeks	0.5
B5	MSC	7	mare	5 weeks	0.9
B6	Control	7	mare	2.5 weeks	1.5
B7	Control	19	gelding	3.5 weeks	7.0
B8	MSC	15	mare	4 weeks	7.7
B9	Control	13	gelding	2.5 weeks	0.5
B10	MSC	9	gelding	2.5 weeks	3.0
Summary					
Median (Mean)	MSC	12 (12)	4 geldings, 3 mares	4 weeks (4 weeks)	2.8 (2.8)
Median (Mean)	Control	16 (14)	4 geldings, 3 mares	2.5 weeks (3 weeks)	1.5 (2.2)

## Data Availability

The datasets used and analyzed during the current study are available from the corresponding author upon reasonable request.

## Supplementary Materials

The following supporting information can be downloaded at: https://www.mdpi.com/article/10.3390/cells12212513/s1, Figure S1: Clinical and imaging parameters displayed as differences over time. Follow-up data were obtained over a time period of 18 months after treatment of early naturally occurring equine tendon lesions with either allogeneic mesenchymal stromal cells (MSCs) in serum or serum alone. Clinical inflammation scores (A) and lameness scores (B) were obtained through blinded clinical examinations. The B-mode ultrasonography score (C), the normalized TSCA (tendon cross-sectional area) (D), the % vascularized area (E), and the normalized % altered tissue (F) were obtained via blinded (semi)quantitative analysis of ultrasound-based imaging (CDU: color Doppler ultrasonography; UTC: ultrasound tissue characterization). To compare the two treatment groups directly, the differences in the respective parameters over time were calculated for each time point (The value before treatment, i.e., at month 0 was substracted from the value at a the respective timepoint of control.), so higher values reflect a worsening effect and lower values indicate improvement. The circles and rhombs mark the median values, error bars, and 95% confidence intervals (A–F). The difference in lameness score at 6 months (B) revealed a significant improvement in the MSC group compared with the serum control group, as marked by the asterisk (*p* < 0.05; Mann–Whitney U test).

## Abbreviations

CDU	Color Doppler ultrasonography
MRI	Magnetic resonance imaging
MSC	Multipotent mesenchymal stromal cell
TCSA	Total cross-sectional area
UTC	Ultrasound tissue characterization
